# Automated AI-based coronary calcium scoring using retrospective CT data from SCAPIS is accurate and correlates with expert scoring

**DOI:** 10.1007/s00330-024-11118-3

**Published:** 2024-10-18

**Authors:** Lilian Henriksson, Mårten Sandstedt, Patrik Nowik, Anders Persson

**Affiliations:** 1 Center for Medical Image Science and Visualization (CMIV), Linköping University, Linköping, Sweden; 2 Unit of Radiology, Department of Health, Medicine and Caring Sciences, Linköping University, Linköping, Sweden; 3 Department of Clinical Science Intervention and Technology, CLINTEC, Karolinska Institutet, Stockholm, Sweden; 4 Siemens Healthineers, Stockholm, Sweden

**Keywords:** Coronary artery disease, Tomography (X-ray computed), Artificial intelligence

## Abstract

**Objectives:**

Evaluation of the correlation and agreement between AI and semi-automatic evaluations of calcium scoring CT (CSCT) examinations using extensive data from the Swedish CardioPulmonary bio-Image study (SCAPIS).

**Materials and methods:**

In total, 5057 CSCT examinations were performed on one CT system at Linköping University Hospital between October 8, 2015, and June 12, 2018. AI evaluations were compared to semi-automatic CSCT results from expert reader evaluations rendered within SCAPIS. Pearson correlation, intraclass correlation coefficients (ICC), and Bland–Altman analysis were applied for Agatston (AS), volume (VS), mass scores (MS), number of lesions and lesion location. Agreement of Agatston score classifications into cardiovascular (CV) risk categories was evaluated with weighted kappa analysis.

**Results:**

The evaluation included 4567 subjects, 2229 (48.8%) male, 2338 (51.2%) female, 50–64 years of age (mean 57.3 ± 4.4). The AS ranged from 0 to 2871 in the cohort, with 2846 subjects having an AS of 0. Mean and median AS were 51.4 and 0.0, respectively. Total AS, VS, MS and number of lesions ICCs were 0.994, 0.994, 0.994, 0.960 (*p* < 0.001), respectively. Bland–Altman analyses rendered mean differences ± 1.96 SD upper and lower limits of agreement for AS −0.04, −52.5 to 52.4, VS −0.44, −46.51 to 45.63, and MS −0.07, −9.62 to 9.48. Weighted kappa analysis for CV risk category classifications was 0.913, and overall accuracy was 91.2%.

**Conclusion:**

There was excellent correlation and agreement between AI and semi-automatic evaluations for all calcium scores, number of lesions and lesion location. High degrees of agreement and accuracy were found for the CV risk categorization.

**Key Points:**

***Question*** *Can AI function as a tool for enhancing the efficiency and accuracy of Coronary Artery Calcium Score (CACS) evaluations in clinical radiology practice?*

***Findings*** *This study confirms the robustness of AI-derived CACS results across extensive datasets, though its generalizability is limited by data acquisition from a single CT system.*

***Clinical relevance*** *This study suggests that AI holds significant promise as a tool for enhancing the efficiency and accuracy of CACS evaluations, with implications for improving patient diagnostics and reducing radiologist workload in clinical practice.*

## Introduction

Non-contrast-enhanced, ECG-triggered coronary calcium scoring computed tomography (CSCT) is a valuable diagnostic tool that identifies coronary artery calcifications (CAC) at minimal radiation exposure [1–6]. This technique has proven reliable in predicting future cardiovascular (CV) events for asymptomatic individuals, independently of conventional risk assessment models [7–10]. Both U.S. [11] and European [12] clinical guidelines recommend CSCT for selected asymptomatic individuals, particularly those with intermediate pre-test clinical CV risk assessments.

The Agatston score (AS), introduced in the early 1990s [13], remains the primary method for coronary artery calcium score (CACS) grading and coronary artery disease (CAD) risk assessment in CSCT [14]. However, alternative techniques such as volume score (VS) and mass score (MS) have been developed to improve the reliability and reproducibility of CACS evaluations [15–17]. Traditionally, expert thoracic radiologists or cardiologists perform this relatively simple task using semi-automatic software, which involves manual identification and marking of CAC lesions. As the global adoption of CSCT continues to rise, there is a growing need for more efficient and automated evaluation systems.

In recent years, significant advancements in artificial intelligence (AI) in radiology have emerged. Several studies have demonstrated AI’s ability to match the diagnostic accuracy of radiologists for CACS at shorter evaluation times [18–20]. This indicates that in the context of CACS, AI offers the prospect of assisting or potentially replacing human readers, presenting an opportunity to alleviate clinical workload and enhance overall efficiency. Testing AI on large datasets is a fundamental step in ensuring that the model is robust, generalizes well to diverse scenarios, and performs reliably in real-world applications. It helps detect and address issues related to bias, variability, and scalability, contributing to the development of trustworthy and effective AI systems.

This study aimed to compare results from a commercially available AI-based CACS software with conventional semi-automatic CACS, using the largest dataset to date acquired from asymptomatic individuals without known CAD. It assessed the correlation and agreement for AS, VS, MS, number of calcified coronary lesions, and lesion location. Additionally, it explored their comparability in categorizing subjects into five CV risk categories.

## Materials and methods

### Study sample

This retrospective, observational, cross-sectional study was conducted in strict adherence to the principles outlined in the Declaration of Helsinki and in accordance with Good Clinical Practice standards. Authorization for this research was granted by the Swedish ethical review authority (Gothenburg Regional Ethical Review Board, DNR 2021-00441) and for the Swedish CArdiopulmonary BioImage Study (SCAPIS) (Gothenburg and Umeå regional ethical review boards, Dnr 2010-228-31M). In compliance with the ethical regulations governing Swedish registries and national legislation, the SCAPIS study subjects were duly informed about their involvement in a registry and provided the option to decline participation or request the removal of their data. The need for written consent was waived for the current study.

All included subjects had undergone CSCT within SCAPIS [21] at Linköping University Hospital between October 8, 2015, and June 12, 2018, amounting to a total of 5057 individuals, 50–64 years of age. Subject data and population demographics (Table 1) were retrospectively gathered from the SCAPIS database. To this date, a total of 45 articles have reported on parts or the entire included study population [22]. None of these articles addressed AI evaluation of CSCT scans.

**Table 1:** Subject Characteristics

Characteristics	Total	Men	Women	Missing values (total)
Sample size, *n*	4567	2229 (48%)	2338 (51.2%)	0
Sociodemographics				
Age, years	57.3 ± 4.4	57.3 ± 4.4	57.4 ± 4.4	0
Anthropometry				
Body mass index, kg/m^2^	26.2 ± 4.4	27.3 ± 4.0	26.4 ± 4.8	0
Smoking status				
Current smoker	419 (9.2%)	197 (8.8%)	222 (9.5%)	75
Former smoker	1438 (31.5%)	664 (29.8%)	774 (33.1%)	
Treatment				
Cholesterol-lowering medication	289 (6.3%)	173 (7.8%)	116 (5.0%)	9
Anti-hypertensive medication	804 (17.6%)	427 (19.2%)	377 (16.1%)	7
Diabetes medication	160 (3.5%)	104 (4.7%)	56 (2.4%)	5
Blood pressure, mm Hg				
Systolic	132.6 ± 17.5	134.2 ± 16.5	130.9 ± 18.2	0
Diastolic	83.4 ± 10.3	83.7 ± 10.0	83.1 ± 10.6	0
Clinical chemistry				
Total cholesterol, mmol/L	5.5 ± 1.0	5.4 ± 1.0	5.7 ± 1.0	0
Glucose, mmol/L	5.8 ± 1.2	5.9 ± 1.3	5.6 ± 1.0	15
HbA1c, mmol/L	36.1 ± 6.7	36.3 ± 7.8	35.8 ± 5.3	11
Estimated GFR, mL/min/1.73 m^2^	86.3 ± 12.3	87.3 ± 11.8	85.3 ± 12.6	0
Risk scores, %				
SCORE	1.5 ± 1.4	1.7 ± 1.5	1.4 ± 1.3	1
Prevalent cardiovascular disease				
Stroke	51 (1.1%)	24 (1.1%)	27 (1.2%)	5
Peripheral artery disease	13 (0.2%)	7 (0.3%)	6 (0.3%)	5
Heredity				
Family history of myocardial infarction (Total/< age 60 years)	1306 (28.6%)/334 (7.3%)	547 (24.5%)/143 (6.4%)	759 (32.5%)/191 (8.2%)	148
Family history of stroke (Total/< age 45 years)	1164 (25.5%)/31 (0.7%)	526 (23.6%)/17 (0.8%)	638 (27.3%)/14 (0.6%)	153

Values are mean ± SD or *n* (%). Smoking status, treatment, prevalent cardiovascular disease, and heredity have been self-reported in the Swedish Cardiopulmonary Bioimage Study questionnaire

*GFR* glomerular filtration rate, *SCORE* Systematic Coronary Risk Evaluation

Exclusion criteria were declination of the CSCT scan (*n* = 45) and those previously established by Wolterink et al [23]. This led to the exclusion of CSCT scans registered as exhibiting severe motion artifacts or high noise levels (*n* = 114), missing images from the CSCT scan (*n* = 1), incomplete scan length coverage (*n* = 4) and missing CACS results tables from the semi-automatic evaluation (*n* = 56). Subjects with a self-reported medical history, including myocardial infarction, angina pectoris, heart failure, heart valve disease, coronary bypass surgery, percutaneous coronary intervention (*n* = 122) or missing information about any of these conditions (*n* = 148), were also excluded to follow guideline recommendations [11, 12] for application of CACS as risk assessment in individuals asymptomatic of CAD.

### CT acquisition parameters and image reconstruction

All CSCT scans were performed using a SOMATOM Definition Flash dual source CT system (Siemens Healthineers). Depending on heart rate and pulse variability, a prospectively ECG-triggered high-pitch spiral (Flash scan) or a sequential scan technique was employed. Both scan protocols had vendor-recommended scan and reconstruction settings, utilizing 120 kVp tube voltage and automated tube current modulation (CARE Dose4D, Siemens Healthineers) set to 80 quality reference mAs. Additional scan parameters included a gantry rotation time of 0.28 s, pitch 3.4, 128 × 0.6 mm collimation. Scan initiation for Flash scans was set at 60% of the cardiac cycle, while sequential scans were conducted at 70%.

Image reconstructions were executed using weighted filtered back projection (WFBP, Siemens Healthineers), a convolution kernel (B35f) dedicated for calcium score reconstructions, 3.0 mm image slice thickness, 1.5 mm increment. Sublingual nitroglycerine and beta-blockers (Metoprolol, i.v. and/or oral) were administered if the heart rate exceeded 60 bpm and no contraindications to these pharmaceuticals were found. Following the CSCT scan, a coronary computed tomography angiography (CCTA) was performed during the same imaging session.

### Data reporting

The semi-automatic CACS evaluations conducted within SCAPIS were made by thoracic radiologists or cardiologists using dedicated CACS post-processing software (syngo.Via VB10A, Siemens Healthineers). All readers had a minimum level 1 training in accordance with the American College of Cardiology Foundation/American Heart Association Clinical Competence Statement on cardiac CT for cardiac CT reading [24] and between 1 and > 10 years’ experience in reading clinical CCTAs. In addition to this, the readers attended yearly training and information sessions within the frame of SCAPIS to assure consistency in reading and reporting.

CACS results charts from the semi-automatic evaluation were saved in the local PACS (Sectra Workstation IDS 7, Sectra). These were pseudonymized and exported for automatic readout. The readout was made with a custom-made MATLAB script (R2023a, Mathworks) that utilized optical character recognition for the automated tabulation. All values were manually controlled. This process rendered access to AS as well as MS and VS, number of lesions and lesion location from the semi-automatic evaluation. No complete pre-control was made for the current study to discover errors in the reference standard results. However, an audit was made of cases misclassified by the AI software by a cardiac imaging radiographer (L.H.) with level 1 training for cardiac CT reading as defined by the Society of Cardiovascular CT [25] and 6 years of experience in cardiac CT research reading. A second read was also performed (by L.H.) on a subset of 200 semi-automatic evaluations acquired consecutively between May 17, 2016, and October 18, 2016, for subsequent reliability analysis.

The automatic evaluation was conducted by exporting the CSCT images from the PACS for post-processing by a newer version of the same CACS post-processing software, which enables AI evaluation (syngo.Via VB60A, Siemens Healthineers) and has been described in previous studies [18, 19, 26]. The charts from this fully automatic evaluation were pseudonymized and exported for automatic readout in the same manner as the semi-automatic evaluation charts and rendered the same type of information.

Based on the AS, CACS values from both AI and semi-automatic evaluations were classified into five commonly used CV risk categories: AS 0 (No identifiable plaque, very low cardiovascular risk), AS 1–10 (Minimal plaque burden, low cardiovascular risk), AS 11–100 (Mild atherosclerotic plaque burden, moderate cardiovascular risk), AS 101–400 (At least moderate atherosclerotic plaque burden, moderately high cardiovascular risk), and AS > 400 (Extensive atherosclerotic plaque burden, high cardiovascular risk) [27].

### Statistical analysis

Continuous data were reported as mean ± standard deviation. Categorical data were presented as counts and percentages. Normality was assumed due to the large sample size (central limit theorem).

Correlation and agreement between the standard reference and the automatic software with respect to the AS, VS, MS, number of lesions and lesion location were evaluated with Pearson correlation (*r*) and intraclass correlation coefficient (ICC). *p*-values < 0.05 were considered statistically significant. Bland–Altman plots were utilized to visualize bias and limits of agreement within a 95% confidence interval. Discrepancies in CV risk classifications were examined through weighted kappa analysis (κ) and accuracy. Kappa coefficients were assessed as 0.01–0.20, slight agreement; 0.21–0.40, fair agreement; 0.41–0.60, moderate agreement; 0.61–0.80, substantial agreement; and 0.81–0.99, almost perfect agreement [28]. The second read subset analysis was conducted with ICC for total and per-vessel AS and weighted kappa analysis (κ) and accuracy for CV risk classification.

Bland–Altman plots were carried out using Microsoft Excel (Microsoft Office 365, MSO, version 2302), while all other statistical analyses were performed using IBM SPSS version 27 (IBM).

## Results

### Study sample

**Fig. 1:**
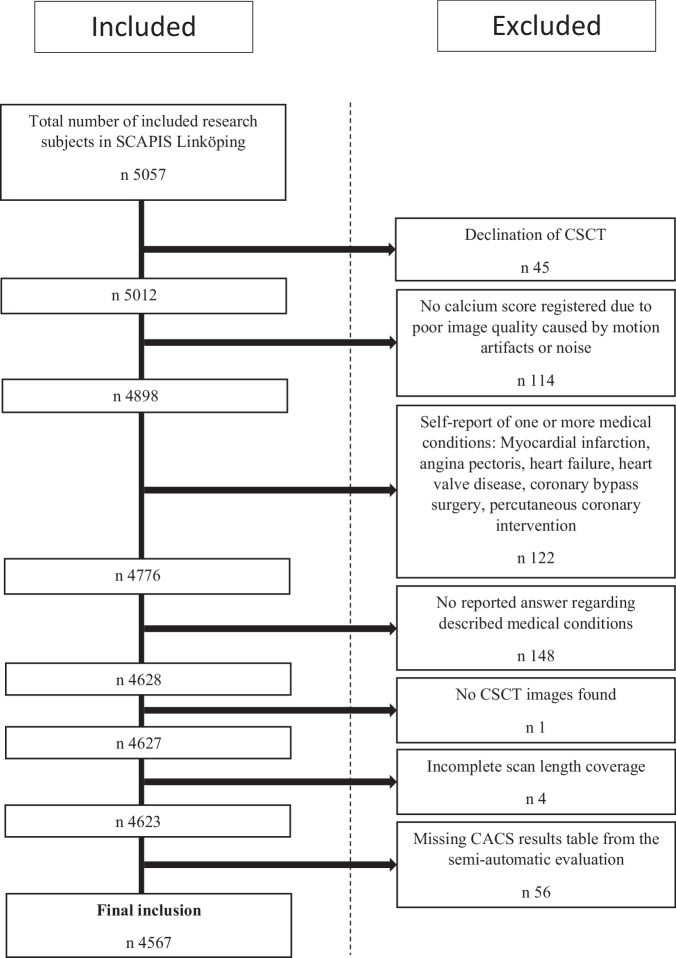
Inclusion and exclusion criteria for semi-automatically derived coronary artery calcium scores (CACS) by trained radiologists or cardiologists from calcium score CT (CSCT) scans performed within the Swedish CArdiopulmonary bio-Image Study (SCAPIS)

Out of 5057 subjects, a total of 4567 (90.3%) were included in the study, meeting the defined inclusion criteria (Fig. 1). Among them, 2229 (48.8%) were male, and 2338 (51.2%) were female. The mean age of the study population was 57.3 ± 4.4 years. Subject characteristics are described in Table 1. Sequential scan technique was used for the CSCT scans in 3313 (72.5%) cases and high-pitch spiral technique was applied in 1254 (27.5%).

### Data reporting

**Fig. 2:**
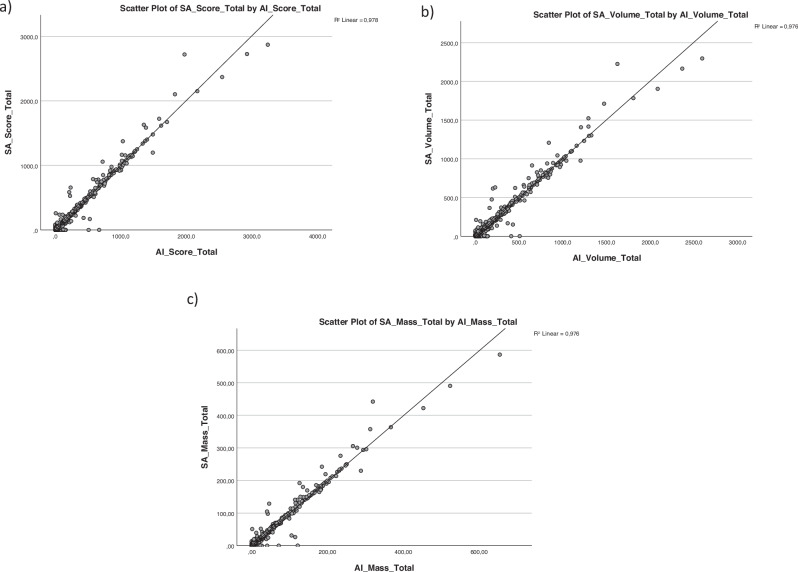
Scatter plots of the Pearson correlation coefficient (*r*) between the AI and semi-automatic evaluations of the (**a**) Agatston score (AS), (**b**) Volume score (VS) and (**c**) Mass score (MS) *r* = 0.989, 0.988 and 0.988, respectively

Pearson correlation (*r*) between the automatic software and the standard reference for the total AS, VS, and MS, yielded correlations with *r* = 0.989, 0.988, and 0.988, respectively (*p* < 0.001) (Fig. 2). Per-vessel correlation for AS was *r* = 0.977 for the left main (LM) combined with left anterior descending (LAD) artery, 0.831 for the circumflex artery (CX) and 0.975 for the right coronary artery (RCA). Differentiation between LM and LAD as lesion locations was not possible in this study as the readers performing the semi-automatic evaluation had been instructed to report all calcifications in these regions as situated in the LAD. VS correlations were *r* = 0.978 (LM + LAD), 0.847 (CX) and 0.970 (RCA), and MS correlations were *r* = 0.979 (LM + LAD), 0.851 (CX) and 0.972 (RCA) (*p* < 0.001).

**Fig. 3:**
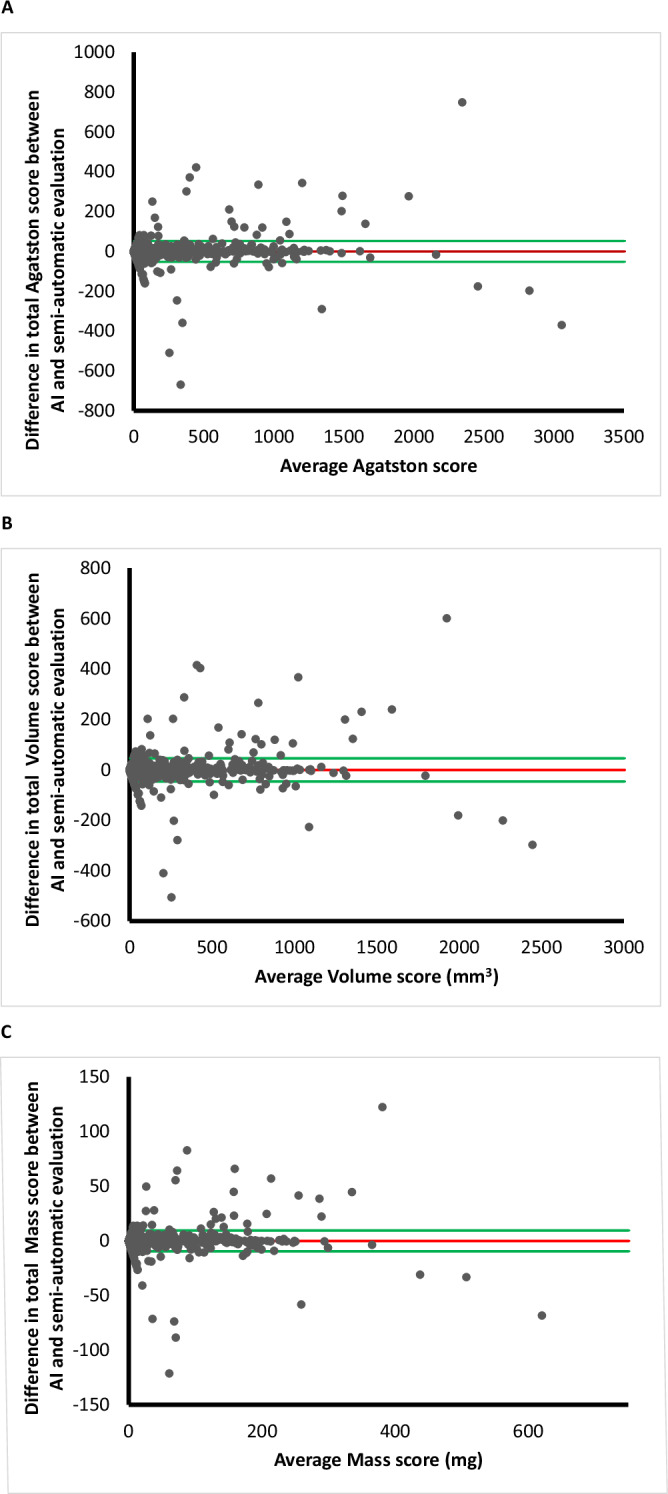
Bland–Altman plots displaying coronary calcium score differences between AI and semi-automatic evaluations in relation to the mean difference between these measurements. **A** Agatston score: mean difference −0.04 and limits of agreement −52.5 to 52.4. **B** Volume score: mean difference −0.44 and limits of agreement −46.51 to 45.63. **C** Mass score: mean difference −0.07 and limits of agreement −9.62 to 9.48

ICCs for the total AS, VS, and MS revealed agreement levels of 0.994, 0.994, and 0.994, respectively. Per-vessel ICC analysis regarding the same metrics respectively resulted in 0.988, 0.989, 0.989 for LM + LAD, 0.904, 0.913, 0.916 for CX and 0.987, 9.985, 0.986 for RCA (*p* < 0.001). ICCs for total number of lesions was 0.960 and for lesion location LM + LAD 0.955, CX 0.881 and RCA 0.918 (*p* < 0.001). Bland–Altman plots displayed mean differences and 1.96 standard deviation upper and lower limits of agreement for AS of −0.04, −52.5 to 52.4, VS of −0.44, −46.51 to 45.63, and MS of −0.07, −9.62 to 9.48 (Fig. 3).

The total AS ranged from 0 to 2871 in the cohort, with 2846 (62.3%) subjects having an AS of 0. Mean and median AS were 51.4 and 0.0, respectively. A confusion matrix for risk category assignment (Table 2) demonstrated an overall accuracy of 91.2% and a weighted kappa analysis (κ) of 0.913 (*p* < 0.001). The CV risk category classification did not correspond between the semi-automatic and automatic evaluations in 402/4567 (8.8%) cases. Out of these, 331/4567 (7.2%) cases were overestimated, and 71/4567 (1.6%) were underestimated by the AI evaluation.

**Table 2:** Confusion matrix with distribution of cardiovascular disease (CVD) risk categorization comparing the standard reference semi-automatic evaluation with the AI software

Risk category changes									
	AI CVD risk								
Standard reference CVD risk	0	1–10	11–100	101–400	> 400	Total	Same	Shift up	Shift down
0	**2544**	266	28	6	2	2846	2544	302	–
1–10	37	**397**	18	1	0	453	397	19	37
11–100	6	13	**724**	8	0	751	724	8	19
101–400	0	1	8	**358**	2	369	358	2	9
> 400	0	0	0	6	**142**	148	142	–	6
Total	2587	677	778	379	146	4567	4165		

Accuracy = 91.2% and weighed kappa analysis (κ) = 0.913 (*p* < 0.001). No risk category shift is indicated by bold numbers. Columns to the right demonstrate a summary of risk category shifting

An audit of all 402 misclassified cases revealed that the semi-automatic evaluation had misregistered or missed calcifications in 80/402 cases (20.1%): Misregistration of image noise or artifacts or non-coronary calcifications in 19/80 (23.75%) cases and missed calcifications in 61/80 (76.25%) cases.

Semi-automatic misregistrations of noise or artifacts led to a one-category CV shift in 10/80 (12.5%) cases (*n* = 10 AS 0 to AS 1–10). Semi-automatic misregistrations of non-coronary calcifications led to one-category CV shift in 5/80 (6.3%) cases (*n* = 1 AS 1–10 to 11–100, *n* = 4 AS 11–100 to 101–400) and a two-category CV shift in 4/80 (5.0%) cases (*n* = 3 AS 0 to 11–100, *n* = 1 AS 1–10 to 101–400). Missed calcifications by the semi-automatic evaluation led to one-category CV shifts in 47/80 (58.8%) cases (*n* = 34 AS 1–10 to AS 0, *n* = 5 AS 11–100 to 1–10, *n* = 8 AS 101–400 to 11–100), two-category CV shifts in 12/80 (15%) cases (*n* = 12 AS 11–100 to 0) and three-category CV shifts in 2/80 (2.5%) cases (*n* = 2 AS 101–400 to 0).

The audit showed that the AI evaluation had misregistered or missed calcifications in 322/402 (80.1%) cases: Misregistration of image noise or artifacts, non-coronary calcifications, and pacemaker electrodes in 270/322 (83.9%) cases and missed calcifications in 52/322 (16.1%) cases.

**Fig. 4:**
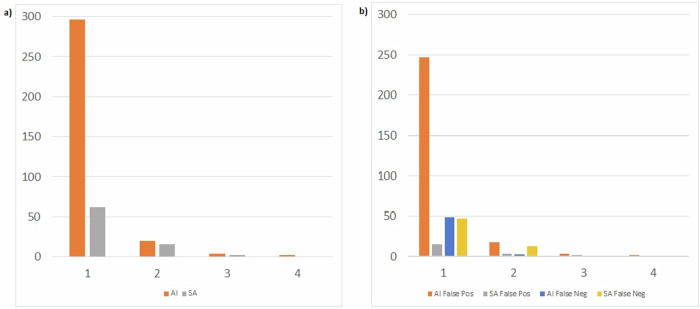
Graphs comparing cardiovascular risk group misclassifications made by the AI and semi-automatic (SA) evaluations causing 1, 2, 3 or 4 risk group shifts. **a** Total number of risk group misclassifications, i.e., combination of both false positives and false negatives. **b** Risk group misclassifications divided into false positive and false negative detection of coronary calcifications for AI and SA evaluation

AI misregistrations of noise or artifacts led to one-category CV shifts in 223/322 (69.3%) cases (*n* = 212 AS 0 to 1–10, *n* = 11 AS 1–10 to 11–100) and two-category CV shifts in 3/322 (0.9%) cases (*n* = 3 AS 0 to 11–100). AI misregistrations of non-coronary calcifications caused CV category shifts in 43/322 (13.4%) cases, a one-category CV shift in 24/322 (7.5%) cases (*n* = 20 AS 0 to 1–10, *n* = 2 AS 1–10 to 11–100, *n* = 2 AS 101–400 to > 400), a two-category CV shift in 14/322 (4.3%) cases (*n* = 13 AS 0 to 11–100, *n* = 1 AS 1–10 to 11–100), a three-category CV shift in 4/322 (1.2%) cases (*n* = 4 AS 11–100 to 0), and a four-category CV shift in 1/322 (0.3%) case (*n* = 1 AS 0 to > 400). AI misregistrations of pacemaker electrodes led to a four-category CV shift in 1/322 (0.3%) case (*n* = 1 AS 0 to > 400). AI missed calcifications led to one-category CV shifts in 49/322 (15.2%) cases (*n* = 34 AS 1–10 to 0, *n* = 5 AS 11–100 to 1–10, *n* = 8 AS 101–400 to 11–100), and two-category CV shifts in 3/322 (0.9%) cases (*n* = 3 AS 11–100 to 0). The audit is presented in Fig. 4.

The sub-analysis of 200 semi-automatic evaluations included 125 AS 0, 24 AS 1–10, 32 AS 11–100 and 12 AS 101–400 and 7 AS > 400 (according to the original CV classification). The ICCs were 1.0 for the total AS and 1.0, 0.999 and 1.0 for LM + LAD, CX and RCA, respectively. Overall accuracy was 98.5% and a weighted kappa analysis (κ) of 0.986 (*p* < 0.001). The semi-automatic evaluation misregistered image noise in two cases, causing CV risk classification shifts from AS 0 to 1–10, and missed a calcification in one case, causing a CV classification shift from AS 11–100 to AS 1–10.

## Discussion

This study used an extensive dataset of 4567 CSCT scans to compare CACS results from an AI software with standard semi-automatic evaluation in asymptomatic individuals without CAD, as recommended by current guidelines [11, 12]. This is, to the best of our knowledge, the largest study so far when it comes to evaluations of AI-derived CACS in non-contrast ECG-gated cardiac scans. The findings showed excellent correlation and agreement between the methods for three types of CAC scores. Per-vessel evaluations and assessments of lesion number and location also demonstrated high agreement. Bland–Altman plots indicated minimal mean differences and narrow limits of agreement for AS, VS, and MS. A minor overestimation bias was seen in the lower CAC scores, which likely is due to the dataset’s skewness, with 62.3% of scans having an AS of 0. Weighted kappa analysis for CV risk category classification showed almost perfect agreement (κ 0.913) with an overall accuracy of 91.2%. Reviewing 402 misclassified CV risk cases revealed the AI evaluations to be correct in 20.1% of these instances. The audit also showed that the semi-automatic evaluation primarily missed calcifications, while the errors by the AI evaluation tended to be misregistrations of noise and artifacts.

Comparisons with other studies in the same field [18, 23, 26, 28–30] are challenging due to variations in study design, inclusion, imaging protocols, reconstruction methods, and quantitative evaluation. Nonetheless, our results for CACS and risk category classification are similar to those in previous studies [18, 23, 25, 29–31], demonstrating excellent correlation, agreement, and subsequent risk classification. The AI software under evaluation here is approved for clinical use and earlier versions have demonstrated its ability to yield reliable CACS evaluation results [26] as well as having a promising time-saving potential [18, 19]. Including a larger number of subjects in this study confirms its robustness across all commonly used CACS metrics. It also indicates the potential of AI in the compilation of extensive databases by facilitating the radiologist’s workload and possibly improving the accuracy of the analysis of both retrospective and prospective datasets. This is underlined by our findings from the audit. A primary AI evaluation followed by expert reader evaluations could reduce misclassification errors especially those causing false negative results. As pointed out previously [18, 26], the AI results should be checked by a human reader.

This study has several limitations, mainly due to its retrospective nature. First, because of the large number of cases, there was no completely controlled reference standard, which has led to an underestimation bias of the AI results. However, all semi-automatic evaluations had been performed by trained readers in a controlled setting regarding interpretation and reporting of CACS and an audit of the misclassifications was also performed to gain further understanding of this matter. Moreover, the second read of a sample of 200 cases showed a high agreement with the original semi-automatic evaluation. Second, the single-center nature of the study means that the CACS test data did not include any variation in regards to the CSCT scan protocol or the CT system used for the CSCT acquisitions. Both AI and expert reader evaluations were also performed on software applications from the same vendor as for the CT system (Siemens Healthineers). This limits the generalizability and transferability of the results. Previous evaluations using CSCT test data acquired on CT systems from different vendors have yielded similar results [29]. Future studies on the accuracy of CACS AI evaluations should include large, more varied test datasets to gain a further understanding of the generalizability of these tools. Third, the per-vessel analysis did not report the calcification burden for LM and LAD separately. As the cardiovascular risk dynamics differ for these vessel regions proper description of the calcification burden here could be of clinical importance [32]. Earlier versions of the same AI software have shown excellent results for reporting LM and LAD CACS separately [19, 26]. Fourth, the included population does not completely align with the recommendations by prevailing guidelines. The age range, for instance, is smaller than the 40–75 years of age recommended by the AHA for CACS in its role to guide pharmacotherapy [11].

In conclusion, this study, using large-scale data of asymptomatic individuals without known CAD, demonstrated excellent correlation and agreement between the automatic software and the reference standard for three types of CACS, the number of lesions and lesion location. Risk category classifications were also highly accurate but with a minor overestimation bias the lower range of CAC scores. This indicates a high level of robustness, and CSCT constitutes an excellent candidate for a comprehensive first assessment by AI.

## Abbreviations

AS: Agatston Score
CACS: Coronary Artery Calcium Score
CAD: Coronary Artery Disease
CSCT: Calcium scoring CT
CV: Cardiovascular
ICC: Intraclass Correlation coefficient
MS: Mass Score
SCAPIS: Swedish CArdioPulmonary bioImage Study
VS: Volume Score
